# Orthorexic Eating Tendencies and Body Shape Concern in Women with Lipedema: A Controlled Cross-Sectional Study

**DOI:** 10.3390/jcm15197678

**Published:** 2026-10-04

**Authors:** Muhsin Doran, Dilara Ekici Zincirci, Ece Güreli Şahan

**Affiliations:** 1 Department of Physical Medicine and Rehabilitation, Liv Hospital Vadistanbul, 34475 Istanbul, Türkiye; gureli4@hotmail.com; 2 Department of Physical Medicine and Rehabilitation, Istanbul Medipol University, 34230 Istanbul, Türkiye; drdilaraekici@gmail.com

**Keywords:** lipedema, orthorexia nervosa, body image, body dissatisfaction, eating behavior, anxiety, depression, women’s health

## Abstract

**Background/Objectives**: Lipedema is an adipose tissue disorder associated with body-related burden. This study compared orthorexic eating tendencies between women with and without lipedema and examined associations with body-shape concern and psychological symptoms. **Methods**: This controlled cross-sectional study included 44 women with lipedema and 44 women without lipedema. Participants completed the Düsseldorf Orthorexia Scale (DOS), Body Shape Questionnaire (BSQ), and Hospital Anxiety and Depression Scale (HADS). Associated variables were examined using multiple linear regression. **Results**: Women with lipedema had higher DOS scores than women without lipedema (25.07 ± 6.55 vs. 20.48 ± 5.05; Cohen’s d = 0.79; *p* < 0.001). BSQ, HADS-Anxiety, and HADS-Depression scores were also higher in the lipedema group (all *p* ≤ 0.001). DOS and BSQ scores were positively correlated in both groups. In the multivariable model, which included group status, age, BMI, BSQ, HADS-A, and HADS-D scores, BSQ score was independently associated with DOS score (B = 0.067; 95% CI 0.025–0.108; *p* = 0.002), whereas group status and the other covariates were not. The model’s adjusted R^2^ was 0.234. **Conclusions**: Women with lipedema exhibited greater orthorexic eating tendencies, body-shape concern, and psychological symptoms than controls. Body-shape concern was independently associated with orthorexic eating tendencies. The group difference in DOS scores is largely explained by the higher BSQ scores accompanying lipedema; this should not be interpreted as indicating that lipedema is unrelated to orthorexic tendencies. These findings support consideration of body image when addressing eating behavior in lipedema care.

## 1. Introduction

Lipedema is a chronic adipose tissue disorder that occurs almost exclusively in women and is characterized by bilateral, symmetrical, and disproportionate accumulation of subcutaneous adipose tissue, typically affecting the lower extremities, with relative sparing of the feet [1]. Altered body proportions may be accompanied by pain, tenderness, easy bruising, a feeling of heaviness in the limbs, and progressive functional limitations [1]. A recent international Delphi consensus process involving experts from 19 countries reaffirmed the clinical definition and management principles of lipedema and reached consensus that the condition may adversely affect mental health and quality of life [2].

Although lipedema is clinically distinct from generalized obesity and lymphedema, it remains underrecognized and is frequently misclassified as obesity [1]. Even physicians from relevant specialties have been reported to have limited knowledge and awareness of the clinical features and treatment of lipedema [3]. Consequently, women with lipedema report prolonged diagnostic delays, difficulties accessing accurate information, and negative healthcare experiences in which their symptoms are attributed to body weight [4]. These experiences may contribute to frustration, reduced trust in healthcare services, and increased psychosocial burden.

Lipedema is associated with impaired health-related quality of life, psychological distress, and body image dissatisfaction [5,6,7]. Greater severity of symptoms such as pain, heaviness, swelling, and limited mobility has also been associated with poorer quality of life and more pronounced depressive symptoms [6,7].

Lipedema-associated adipose tissue is difficult to reduce through diet, exercise, or bariatric surgery [1]. The combination of visible and persistent disproportion, repeated weight-focused advice, and the limited response of affected areas to conventional weight-loss approaches may create conditions conducive to maladaptive eating behaviors. It has been suggested that some women may engage in restrictive eating, overeating, or other maladaptive eating behaviors to cope with body-related distress and negative emotions [8,9]. Dietary strategies adopted to control pain, edema, inflammation, body weight, or disease progression may evolve into rigid and excessively restrictive eating rules, particularly amid nonstandardized advice disseminated through social media and patient communities [10], where content popularity may not reflect quality and professionally produced content tends to be more reliable [11].

Direct evidence on eating behavior in lipedema is limited. In an online study involving 47 women recruited through a lipedema community, approximately two-thirds of participants scored at or above the screening threshold on the Eating Attitudes Test [12]. However, recruitment from an online community, partial reliance on self-reported diagnoses, and the absence of a control group limit the interpretability of these findings. Studies are therefore needed that compare women with clinically diagnosed lipedema with an appropriate control group and assess specific dimensions of eating behavior alongside body image and psychological symptoms.

One relevant behavior in this context is orthorexic eating. An interest in healthy eating is not, by itself, indicative of a problem. Orthorexia nervosa is characterized by an excessive preoccupation with foods perceived as healthy or pure, rigid self-imposed dietary rules, and marked distress when these rules are violated [13,14,15]. The Düsseldorf Orthorexia Scale (DOS) was developed to assess orthorexic eating behavior along a continuous dimension of severity rather than as a categorical diagnosis [16]. Body image may represent a potential link between lipedema and orthorexic eating. In nonclinical samples, orthorexic tendencies have been shown to be associated with body image and perfectionism [17]; orthorexia nervosa symptoms have also been reported to be prospectively associated with subsequent eating disorder and depressive symptoms [18]. Lipedema leads to persistent and often pronounced body disproportion that is not fully captured by body mass index (BMI); therefore, two women with similar BMI values may differ substantially in body dissatisfaction, perceived stigma, and pressure to change their appearance [19,20]. Anxiety and depressive symptoms may heighten dietary hypervigilance or reinforce rigid behaviors adopted to regain a sense of control [21,22].

It remains unknown whether women with lipedema exhibit greater orthorexic eating tendencies than women without lipedema and which factors are associated with these tendencies. We hypothesized that women with lipedema would exhibit greater orthorexic eating tendencies and body shape concern. The main objectives were to compare orthorexic eating tendencies between women with and without lipedema and to examine the associations of these tendencies with body shape concern, anxiety, and depressive symptoms. Additional objectives were to compare body shape concern, anxiety, and depressive symptoms between the groups.

## 2. Materials and Methods

### 2.1. Study Design and Reporting

This single-center, observational, cross-sectional study compared women with clinically diagnosed lipedema with women without lipedema. The study was conducted at Istanbul Medipol University Hospital; participant recruitment and data collection took place from 30 June to 27 August 2026. Reporting followed the Strengthening the Reporting of Observational Studies in Epidemiology (STROBE) statement for cross-sectional studies [23].

### 2.2. Participants and Eligibility Criteria

Women aged 18–65 years who presented to the Physical Medicine and Rehabilitation outpatient clinic and met the eligibility criteria were included in the study. Inclusion criteria common to both groups were the ability to understand and complete questionnaires in Turkish and the provision of written informed consent. An additional inclusion criterion for the lipedema group was a clinical diagnosis of lipedema. Additional criteria for the control group were no previous diagnosis of lipedema and no clinical findings suggestive of lipedema according to the screening method used in the study. All diagnostic assessments were performed by a physical medicine and rehabilitation specialist with eight years of active clinical experience in the assessment, follow-up, and treatment of patients with lipedema. Clinical eligibility and diagnostic assessments were completed before questionnaire administration, and the assessor was blinded to participants’ questionnaire responses.

Lipedema was diagnosed through a structured clinical history and physical examination based on prespecified clinical criteria derived from established sources [1,24]. The assessment included inspection of fat distribution and symmetry; examination for relative sparing of the hands and feet; palpation of the subcutaneous tissue for nodularity and fibrotic changes; assessment of pain or tenderness in the affected areas; and history-taking regarding easy bruising and the response of regional fat deposits to previous weight-loss attempts. Women were classified as having lipedema when all applicable predefined clinical features were present.

Generalized obesity was differentiated from lipedema by assessing whether adipose tissue distribution was generalized and proportionate or predominantly symmetrical and disproportionate in the extremities, together with the presence or absence of hand and foot sparing, pain or tenderness, easy bruising, and palpable subcutaneous nodularity.

Differential assessment for secondary lymphedema and venous disease was based on clinical history and physical examination. For secondary lymphedema, the history included previous lymph-node surgery or radiotherapy, malignancy, recurrent cellulitis or erysipelas, trauma, vascular surgery, the timing and laterality of swelling, and changes in swelling during the day. Examination included assessment of dorsal foot and toe involvement, the Stemmer sign, pitting edema, skin thickening or fibrosis, and marked asymmetry. Venous assessment included a history of swelling aggravated by prolonged standing and relieved by elevation or overnight rest, leg heaviness, cramping, pruritus, varicose veins, and previous deep-vein thrombosis. Examination included assessment for varicose veins, ankle edema, hemosiderin-related pigmentation, stasis dermatitis, lipodermatosclerosis, and healed or active venous ulcers. When the history or examination raised clinical uncertainty, appropriate imaging was requested according to the suspected differential diagnosis. When venous reflux or obstruction was suspected, duplex venous ultrasonography was requested.

Control participants were recruited from hospital staff, patients’ relatives, and accompanying caregivers. All control participants underwent clinical history-taking and physical examination by the same physician. The assessment included fat distribution and symmetry, relative sparing of the hands and feet, subcutaneous nodularity, pain or tenderness, easy bruising, and findings suggestive of lymphedema or venous disease. Women with clinical findings suggestive of lipedema were not included in the control group. During recruitment, care was taken to ensure that the age and body mass index (BMI) distributions of the control group were broadly similar to those of the lipedema group.

Exclusion criteria were pregnancy; active malignancy; secondary lymphedema; lipolymphedema; surgery or liposuction for lipedema within the previous six months; advanced cardiac, pulmonary, renal, or hepatic failure; cognitive impairment preventing questionnaire completion; an active psychotic disorder; and a previously diagnosed eating disorder. Records with missing or internally inconsistent data that precluded reliable calculation of the main outcome measures were also excluded.

The original a priori calculation indicated a minimum total sample size of 46 participants. Recruitment was continued beyond this minimum to increase the sample size and obtain balanced study groups. Enrollment was subsequently concluded after 88 eligible participants had been included, comprising 44 participants with lipedema and 44 controls. No formal precision-based or regression-specific stopping rule had been prespecified.

### 2.3. Data Collection and Study Procedure

After eligibility assessment and provision of written informed consent, participants completed a standardized data collection form and the study questionnaires in a quiet setting. Completion of the questionnaires took approximately 20–30 min. Participants received no financial incentives. Standardized information on current dietary patterns, recent weight-loss attempts, previous nutritional treatment or dietitian support, exercise behavior, and exposure to diet-related information through social media or lipedema patient communities was not collected.

### 2.4. Primary Outcome Measure

#### Düsseldorf Orthorexia Scale

Orthorexic eating tendencies were assessed using the Turkish version of the Düsseldorf Orthorexia Scale (DOS), a 10-item self-report instrument developed to assess orthorexic eating behavior [16]. Each item is rated on a four-point Likert scale, yielding a total score ranging from 10 to 40, with higher scores indicating greater orthorexic eating tendencies. The Turkish version has been evaluated in a clinical sample and reported to demonstrate adequate psychometric properties [25,26].

The DOS total score was treated as a continuous variable and designated as the primary outcome measure. The scale was used to assess symptom severity and was not interpreted as a diagnostic tool for orthorexia nervosa. No cutoff-based classification was used in the inferential analyses.

### 2.5. Secondary Outcome Measures

#### 2.5.1. Body Shape Questionnaire

Body shape concerns were assessed using the 34-item Body Shape Questionnaire (BSQ), developed by Cooper et al. [27]. The questionnaire assesses body shape-related concern and distress, fear of weight gain, body dissatisfaction, and excessive preoccupation with appearance over the preceding four weeks. Items are rated on a six-point Likert scale, yielding a total score ranging from 34 to 204, with higher scores indicating greater body shape concern. The Turkish version of the scale has previously been evaluated [28]. The BSQ total score was analyzed as a continuous variable.

#### 2.5.2. Hospital Anxiety and Depression Scale

Anxiety and depressive symptoms were assessed using the Hospital Anxiety and Depression Scale (HADS) [29]. The scale comprises 14 items divided into two seven-item subscales assessing anxiety (HADS-A) and depression (HADS-D). Each item is scored from 0 to 3, and total scores for each subscale range from 0 to 21, with higher scores indicating greater symptom severity. The validity and reliability of the Turkish version have been demonstrated [30]. The anxiety and depression subscale scores were analyzed separately as continuous variables. The scale was used to assess psychological symptom burden rather than to establish psychiatric diagnoses.

#### 2.5.3. Pain Intensity

Average lower-extremity pain intensity over the preceding week was assessed using a 10 cm visual analog scale (VAS), with 0 indicating “no pain” and 10 indicating “the worst pain imaginable” [31].

#### 2.5.4. Clinical Characteristics of Lipedema

Lipedema anatomical type was determined clinically according to the distribution-based classification described by Herbst et al. and Buso et al. [1,24]. Type I was defined as involvement predominantly affecting the hips and buttocks; type II as involvement extending from the hips to the knees; type III as involvement extending from the hips to the ankles; type IV as upper-extremity involvement, which could coexist with a lower-extremity type; and type V as isolated or predominant involvement of the lower legs. Participants were classified according to their predominant lower-extremity distribution. Upper-extremity and lower-leg distribution patterns were also systematically assessed; however, no participant exhibited type IV involvement or an isolated or predominant type V distribution.

Morphological stage was determined according to the clinical descriptions provided by Herbst et al. and Buso et al. [1,24]. Stage 1 was defined by a smooth skin surface with thickened subcutaneous tissue and palpable small nodules; stage 2 by an uneven or dimpled skin surface with more pronounced nodular and fibrotic changes; and stage 3 by large nodules, overhanging tissue lobules, or marked contour deformity [1,24]. Participants with clinically evident concomitant lymphedema, described as lipolymphedema or stage 4 by Buso et al. [24], were excluded according to the study protocol. All anatomical typing and morphological staging assessments were performed by the same clinician.

Disease duration, family history, and previous lipedema treatments were recorded based on participant self-report and cross-checked against clinical records whenever possible. Disease duration was defined as the time elapsed since the clinical diagnosis of lipedema and was categorized as newly diagnosed, <1 year, 1–3 years, 3–5 years, or >5 years.

### 2.6. Sample Size

The original a priori sample-size calculation was based on the anticipated association between orthorexic eating tendencies and body shape concerns. A previous study using both the Düsseldorf Orthorexia Scale (DOS) and the Body Shape Questionnaire (BSQ) reported a correlation of r = 0.61 between the two measures [32]. To adopt a more conservative assumption, an expected correlation of r = 0.40 was used. Using G*Power version 3.1.9.7 (correlation: bivariate normal model), with a two-sided α level of 0.05 and 80% power, the minimum required total sample size was 46 participants. The final analytic sample comprised 88 participants (44 women with lipedema and 44 controls). Because the original a priori calculation was based on the planned correlational analysis rather than specifically on the between-group comparison of DOS scores, an additional sensitivity analysis was performed to characterize the detectable effect size for the primary between-group comparison using the achieved sample size. With 44 participants per group, a two-sided α level of 0.05, and 80% power, the achieved sample was sufficient to detect an effect size of Cohen’s d = 0.60 or greater using an independent-samples *t*-test.

### 2.7. Statistical Analysis

The between-group comparison of DOS scores was designated as the primary analysis. Between-group comparisons of BSQ, HADS-A, and HADS-D scores were considered secondary analyses, whereas correlation, interaction, multivariable regression, and additional sensitivity analyses were considered exploratory. No formal adjustment for multiple comparisons was applied to the secondary or exploratory analyses; therefore, their *p* values were interpreted as supportive and exploratory rather than confirmatory. For these analyses, emphasis was placed on effect estimates and 95% confidence intervals rather than statistical significance alone.

Statistical analyses were performed using IBM SPSS Statistics for Windows, version 27.0 (IBM Corp., Armonk, NY, USA). The distribution of continuous variables was assessed using visual methods (histograms and Q–Q plots) together with the Shapiro–Wilk test. Continuous variables are presented as mean ± standard deviation (SD); for age and BMI, which showed deviations from normality, median and interquartile range (IQR) are additionally reported. Categorical variables are expressed as frequency and percentage.

Baseline demographic and clinical characteristics were compared between the lipedema and control groups using the independent-samples *t*-test or Mann–Whitney U test for continuous variables and the chi-square test or Fisher’s exact test for categorical variables, as appropriate. The choice between parametric and non-parametric tests was based on the distributional assessment of the respective variables. Specifically, age and BMI showed deviations from normality on the Shapiro–Wilk test in both groups and were therefore compared using the Mann–Whitney U test. Between-group differences in continuous outcomes were reported together with mean differences, 95% confidence intervals (95% CIs), and Cohen’s d effect sizes.

Pearson’s correlation analysis was performed separately within each group to evaluate the relationships between DOS scores and age, BMI, BSQ, HADS-A, and HADS-D scores. Group-specific analyses were used to characterize within-group associations and to avoid conflating within-group relationships with between-group differences in score distributions. For the BSQ–DOS correlations, 95% CIs were estimated using 5000 bootstrap resamples, and the correlation coefficients between the two groups were compared using Fisher’s r-to-z transformation. To formally assess whether the association between BSQ and DOS differed by group, a pooled linear regression model including group status (control = 0, lipedema = 1), mean-centered BSQ score, and a group × BSQ interaction term was fitted.

Sequential linear regression models were used to distinguish the unadjusted association between group status and DOS from associations conditional on demographic and psychological characteristics. DOS score was entered as the dependent variable in all models. Model 1 included group status alone (control = 0, lipedema = 1). Model 2 additionally included age and BMI to estimate the group association conditional on these demographic characteristics. In exploratory Model 3, BSQ, HADS-A, and HADS-D scores were additionally entered to examine the group association conditional on concurrently measured psychological characteristics. All predictors were entered simultaneously using the enter method within each model. Because BSQ and HADS scores were measured concurrently with DOS and may represent correlates or potentially lie on pathways linking lipedema status with eating behavior, Model 3 was interpreted as an exploratory conditional model and not as an estimate of mediation or of the total association between lipedema status and DOS.

The assumptions of linear regression were evaluated using residual diagnostic plots and influence statistics. Linearity and homoscedasticity were assessed by plotting standardized residuals against standardized predicted values, and residual normality was evaluated using histograms and normal P–P plots. Multicollinearity was assessed using tolerance and variance inflation factor (VIF) values. Potentially influential observations were evaluated using studentized residuals, leverage values, Mahalanobis distance, and Cook’s distance.

Because education and marital status differed between groups, an additional sociodemographic sensitivity model was fitted by adding these variables to exploratory Model 3. As a further sensitivity analysis of the stability of the Model 3 coefficient estimates, bootstrap estimates based on 5000 resamples were obtained using bias-corrected and accelerated (BCa) 95% CIs.

Additional exploratory analyses included descriptive evaluation of conventional DOS cut-offs, correlations of pain intensity with DOS and BSQ within the lipedema group, and a between-group DOS comparison restricted to participants with university/postgraduate education. All statistical tests were two-sided, with *p* < 0.05 used as the nominal threshold for statistical significance. For secondary and exploratory analyses, however, *p* values were interpreted in the context of the prespecified analysis hierarchy and without formal multiplicity adjustment.

### 2.8. Ethical Considerations

The study was approved by the Istanbul Medipol University Non-Interventional Clinical Research Ethics Committee on 25 June 2026 (approval no. 950). Written informed consent was obtained from all participants before enrollment. The study was conducted in accordance with the principles of the Declaration of Helsinki. Participant data were anonymized before statistical analysis, and no personally identifiable information was included in the research dataset.

## 3. Results

### 3.1. Participant Characteristics

**Figure 1:**
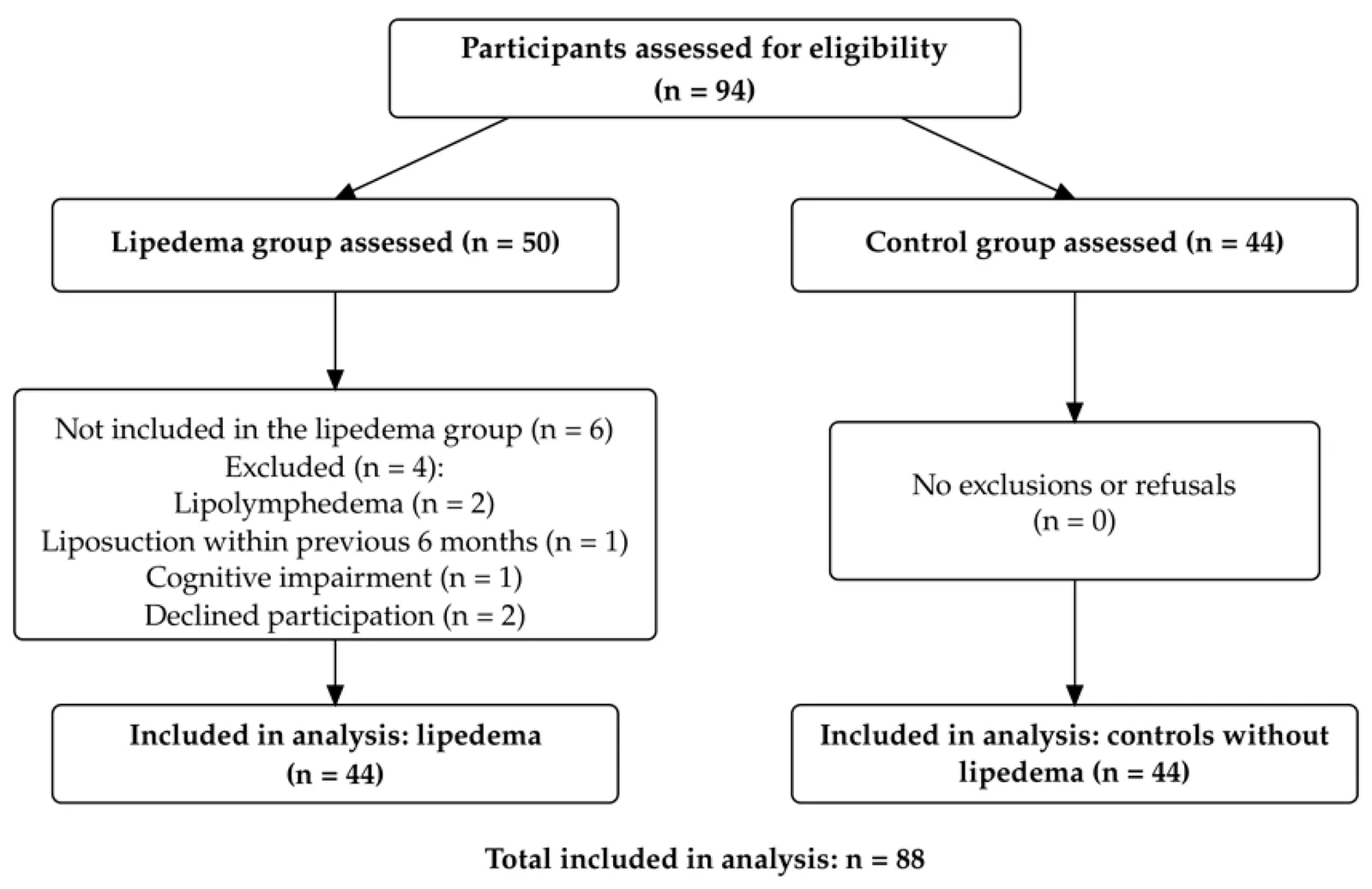
Participant flow diagram.

A total of 94 women were screened for participation. Four women were excluded because they met the exclusion criteria: two had lipolymphedema, one had undergone liposuction for lipedema within the previous six months, and one had cognitive impairment that prevented reliable questionnaire completion (Figure 1). Two additional women declined to participate (Figure 1). Consequently, 88 women, comprising 44 women with lipedema and 44 controls without lipedema, were included in the final analysis. No participants were excluded after enrollment, and complete data were available for all primary and secondary outcome measures. The groups were comparable in terms of age, body mass index (BMI), occupation, smoking status, alcohol consumption, comorbidity, regular medication use, and previous psychiatric diagnosis (all *p* > 0.05). No participant was taking psychotropic medication at the time of assessment; therefore, this variable was not examined as a covariate. However, women with lipedema were more likely to be married (*p* = 0.013) and had lower educational attainment compared with controls without lipedema (*p* = 0.007) (Table 1).

### 3.2. Clinical Characteristics of the Lipedema Group

The mean pain intensity among patients with lipedema was 5.57 ± 2.24 on the VAS. Most patients had a disease duration of 1–3 years (43.2%), followed by newly diagnosed or <1-year disease duration (34.1%). Type III lipedema (54.5%) and stage 2 disease (54.5%) were the most common clinical presentations. Half of the participants (50.0%) reported a positive family history of lipedema, whereas 27.3% were unable to determine whether the disease was present among their relatives. The majority of patients (88.6%) had not received previous treatment for lipedema (Table 2).

### 3.3. Between-Group Comparison of Orthorexic Eating Tendencies, Body Shape Concern, Anxiety, and Depressive Symptoms

For the primary outcome, DOS scores were higher in participants with lipedema than in controls without lipedema (25.07 ± 6.55 vs. 20.48 ± 5.05; mean difference 4.59, 95% CI 2.11–7.07; Cohen’s d = 0.79). Descriptively, DOS scores ≥ 25 were observed in 23/44 (52.3%) participants with lipedema and 9/44 (20.5%) controls, while scores ≥ 30 were observed in 14/44 (31.8%) and 2/44 (4.5%), respectively. The mean between-group difference in DOS corresponded to approximately 15% of the scale’s total possible score range (10–40). As a validated minimal clinically important difference for the DOS has not been established, this proportion is provided to contextualize the magnitude of the observed difference rather than to define a threshold for clinical importance.

In the secondary analyses, participants with lipedema had higher BSQ (111.89 ± 33.26 vs. 81.20 ± 32.00), HADS-A (9.34 ± 3.48 vs. 6.66 ± 3.71), and HADS-D (7.50 ± 3.65 vs. 4.39 ± 2.64) scores than controls. The effect sizes for these secondary between-group differences ranged from Cohen’s d = 0.75 to 0.98 (Table 3). As no multiplicity adjustment was applied to the secondary outcomes, these findings should be interpreted as supportive rather than confirmatory.

In a further sensitivity analysis restricted to participants with university/postgraduate education, the between-group difference in DOS was retained (24.64 ± 6.24 vs. 20.40 ± 4.98; mean difference 4.23, 95% CI 1.65–6.81; *p* = 0.002).

Observed score ranges in the overall sample were 10–39 for DOS, 34–172 for BSQ, 0–15 for HADS-A, and 0–19 for HADS-D. Score distributions showed no marked skewness (overall skewness: DOS = 0.250, BSQ = 0.044, HADS-A = −0.110, and HADS-D = 0.695). Floor observations were uncommon, occurring in 1.1%, 3.4%, 1.1%, and 5.7% of participants for DOS, BSQ, HADS-A, and HADS-D, respectively, and no participant reached the theoretical maximum of any scale. No substantial floor or ceiling effects were apparent. Group-specific observed ranges and distributional indices were also examined and did not indicate marked distributional abnormalities.

### 3.4. Variables Associated with Orthorexic Eating Tendencies

**Figure 2:**
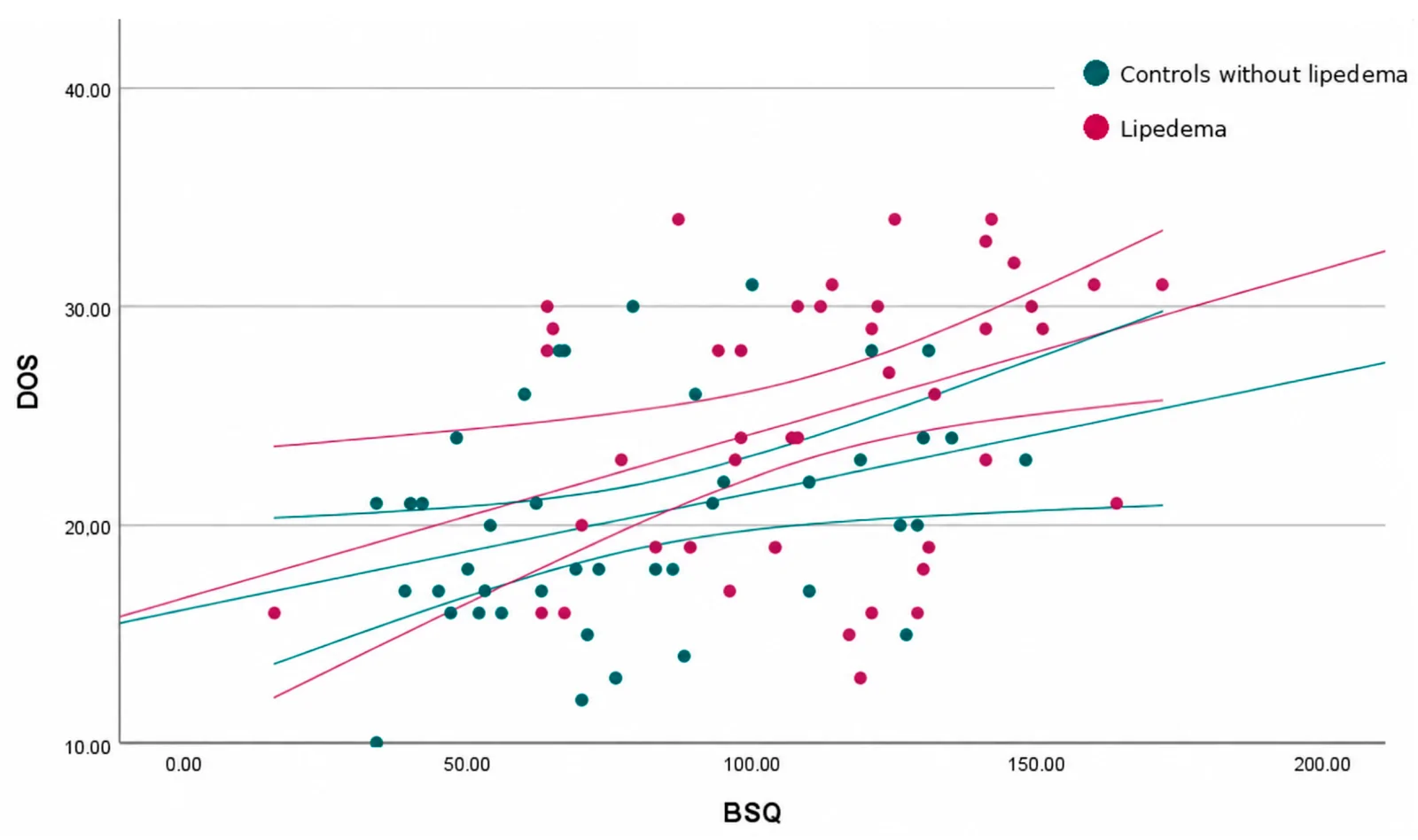
Association between Body Shape Questionnaire (BSQ) and Düsseldorf Orthorexia Scale (DOS) scores according to group. Solid lines represent fitted linear regression lines, and the outer lines represent 95% confidence bands.

DOS scores were positively correlated with BSQ scores in both controls without lipedema (r = 0.339, 95% bootstrap CI 0.103–0.548; *p* = 0.024) and participants with lipedema (r = 0.382, 95% bootstrap CI 0.089–0.607; *p* = 0.011) (Figure 2). No significant correlations were observed between DOS scores and age, BMI, HADS-A, or HADS-D in either group (all *p* > 0.05) (Table 4). Fisher’s r-to-z comparison provided no evidence of a difference in the strength of the BSQ–DOS association between the groups (z = −0.22, *p* = 0.82). Consistent with this finding, a pooled linear regression model including group, mean-centered BSQ, and the group × BSQ interaction showed no evidence of effect modification by group (interaction B = 0.022, 95% CI −0.051 to 0.094; *p* = 0.553). In exploratory analyses within the lipedema group, pain intensity was not significantly correlated with DOS (r = 0.151, 95% bootstrap CI −0.134 to 0.423; *p* = 0.326) or BSQ (r = 0.254, 95% bootstrap CI −0.034 to 0.576; *p* = 0.096).

### 3.5. Factors Associated with Orthorexic Eating Tendencies

Sequential regression models were used to distinguish the unadjusted between-group association from associations conditional on demographic and psychological variables. In Model 1, which included group status alone, participants with lipedema had DOS scores that were 4.591 points higher than those of controls without lipedema (B = 4.591, 95% CI 2.114–7.068; *p* < 0.001). In Model 2, after conditioning on age and BMI, the group coefficient remained similar (B = 4.150, 95% CI 1.656–6.644; *p* = 0.001). Model 2 explained 17.6% of the variance in DOS scores (adjusted R^2^ = 0.146; F(3,84) = 5.971; *p* = 0.001).

In the exploratory Model 3, BSQ, HADS-A, and HADS-D scores were additionally included to examine the group association conditional on concurrently measured psychological characteristics. The overall model was statistically significant (F(6,81) = 5.435, *p* < 0.001), explaining 28.7% of the variance in DOS scores (adjusted R^2^ = 0.234). In this model, the group coefficient was smaller and its confidence interval included zero (B = 1.875, 95% CI −0.923 to 4.672; *p* = 0.186), whereas BSQ was positively associated with DOS (B = 0.067, 95% CI 0.025–0.108; standardized β = 0.384; *p* = 0.002). Age, BMI, HADS-A, and HADS-D were not statistically significantly associated with DOS (Table 5). Because BSQ and psychological symptoms were measured concurrently with DOS, Model 3 represents a conditional association and should not be interpreted as evidence that body-shape concern mediates the overall association between lipedema status and DOS.

Regression diagnostics for the fully adjusted model did not indicate substantial violations of linearity, residual normality, or homoscedasticity. There was no evidence of problematic multicollinearity (VIF range, 1.160–2.018; tolerance range, 0.496–0.862). Studentized residuals ranged from −2.56 to 1.99, and the maximum Cook’s distance was 0.086, indicating no strongly influential observations. Bootstrap sensitivity analysis using 5000 resamples yielded results consistent with the conventional estimates: BSQ remained positively associated with DOS (B = 0.067, BCa 95% CI 0.025–0.102; *p* = 0.002), whereas the BCa confidence interval for the group coefficient included zero (B = 1.875, BCa 95% CI −0.774 to 4.840; *p* = 0.186).

In an additional sociodemographic sensitivity analysis, education and marital status were added to Model 3. The overall pattern of findings was unchanged. BSQ remained positively associated with DOS (B = 0.075, 95% CI 0.032–0.118; *p* = 0.001), while the group coefficient remained statistically nonsignificant (B = 1.644, 95% CI −1.331 to 4.620; *p* = 0.275). Neither education nor marital status was significantly associated with DOS (both *p* > 0.20).

## 4. Discussion

In this controlled cross-sectional study, women with clinically diagnosed lipedema exhibited greater orthorexic eating tendencies than women without lipedema; they also had higher levels of body shape concern, anxiety, and depressive symptoms. In both groups, orthorexic eating tendencies were associated with body shape concern but not with age, BMI, anxiety, or depressive symptoms. When all variables were considered together, body shape concern was the only variable independently associated with orthorexic eating tendencies.

Studies directly examining eating behavior in lipedema are scarce. In Kunzová’s online study of 47 women, disordered eating was reported in approximately two-thirds of women with lipedema; however, lipedema diagnosis relied on self-report, without clinical confirmation, and the study lacked a control group [12]. In the orthorexia literature, a meta-analysis reported a moderate association between orthorexic symptoms and general eating disorder symptoms [33]. Orthorexic tendencies have also been investigated in other clinical populations in which diet plays a prominent role in disease management; Gercek et al. found higher levels of orthorexic symptoms in adolescents with celiac disease than in controls [34]. In our study, women with clinically diagnosed lipedema exhibited greater orthorexic eating tendencies than controls without lipedema. This finding suggests that, in addition to previously reported disordered eating behaviors, orthorexic tendencies characterized by rigid, health-focused dietary rules may also be more pronounced in women with lipedema. This interpretation is consistent with the clinical view that women with lipedema may use eating behavior to cope with body-related distress [8]. However, the present study did not assess dietary practices, motivations for food choice, recent weight-loss attempts, or eating in response to body-related distress. Therefore, it cannot identify the behavioral processes underlying the higher DOS scores observed in the lipedema group. Future studies could examine whether metacognitive beliefs and cognitive rigidity are relevant to eating-related experiences in women with lipedema [35].

In a recent study by Goodrose-Flores et al. body shape concern assessed using the BSQ was higher in women with lipedema than in healthy controls; the presence of lipedema remained associated with greater odds of being in a higher BSQ category after adjustment for age and BMI [36]. Examining the relationship between body image and orthorexic symptoms, Liu et al. reported that higher baseline body appreciation predicted lower levels of orthorexic symptoms at follow-up, although the effects were small [37]. Abou Tayeh et al. found a negative association between the ability to accept negative body-related thoughts and feelings and DOS scores [38], whereas Barnes and Caltabiano reported that orthorexic tendencies were associated with body image and perfectionism [17]. These studies examined different, although related, aspects of body-related experience, including appearance-related body image, body appreciation, and acceptance of body-related thoughts and feelings [17,37,38]. In our study, DOS scores were positively associated with BSQ-measured body-shape concern in both groups. In the multivariable model, body shape concern was the only variable independently associated with DOS scores, whereas group membership was no longer independently associated. Statistically, this pattern indicates that body shape concern accounted for the group difference in orthorexic tendencies; however, given the cross-sectional design, it does not establish temporal order, mediation, or causality.

Lipedema is characterized by disproportionate accumulation of adipose tissue in the extremities relative to the trunk, and this regional difference in body composition may not be adequately captured by BMI. In this study, the higher BSQ scores in the lipedema group despite similar BMI values between groups suggest that body shape concerns may not be explained solely by total body weight. This interpretation is supported by previous studies reporting greater body image concerns in women with lipedema independently of BMI and showing that, among individuals with obesity and similar body composition, the presence of lipedema is associated with greater body image dissatisfaction [19,36,39,40].

In our study, anxiety and depressive symptoms were higher in the lipedema group. This finding is consistent with studies reporting psychological distress and impaired quality of life in lipedema [5,6,7] and with the consensus view that mental health may be adversely affected [2]. However, findings regarding the associations of orthorexic tendencies with anxiety and depression are inconsistent. Sezer Katar et al. reported small positive correlations [41], whereas the clinical validation study of the Turkish DOS found no significant associations between DOS scores and Hamilton anxiety and depression scores [25]. Our finding of neither significant correlations between HADS subscale scores and DOS scores nor independent associations in the multivariable model is consistent with the Turkish DOS study. Differences in measurement instruments, the clinical characteristics of the samples, and sample sizes may have contributed to the discrepant findings across studies. In particular, the small correlations reported in the previous study suggest that these associations may have gone undetected in the present sample. Furthermore, cross-sectional findings do not rule out associations that may develop over time; indeed, a longitudinal study reported that orthorexic symptoms predicted subsequent eating disorder and depressive symptoms [18].

Distinguishing an ordinary interest in healthy eating from rigid and restrictive eating patterns that may impair functioning is important when translating these findings into clinical assessment and care. The study by Lasson et al. supports distinguishing healthy orthorexia from pathological orthorexic features [14], while expert consensus emphasizes the importance of distress and functional impairment in assessment [13]. Levinson et al. also reported that dichotomous thinking that categorizes foods strictly as “good” or “bad” is associated with body dissatisfaction and various disordered eating behaviors [42]. The S2k guideline for lipedema likewise recommends assessment for suspected eating disorders and the provision of nutritional therapy alongside psychological support [43]. The elevated DOS scores and their associations with BSQ scores in our study support investigating the inclusion of body shape concern and the flexibility of eating rules in nutritional assessment and counseling within lipedema care. However, DOS scores reflect self-reported orthorexic eating tendencies on a dimensional screening scale and should not be interpreted as establishing a clinical disorder or, by themselves, as indicating a need for treatment.

The strengths of this study include a lipedema diagnosis based on clinical assessment by an experienced physician, clinical assessment for lipedema in the control group, and evaluation of both groups during the same period. Assessing orthorexic tendencies alongside body shape concern and psychological symptoms allowed the examination of associated variables beyond between-group comparisons. Excluding participants with a previously diagnosed eating disorder or an active psychotic disorder limited the potential influence of known severe psychiatric conditions on the findings.

Several limitations of this study should be considered. First, the cross-sectional design does not permit inferences about causality or temporal ordering; it cannot establish whether body shape concern precedes orthorexic eating, follows it, or whether both arise from shared factors. Second, the study was conducted at a single center; participants with lipedema were recruited consecutively, whereas controls were recruited from hospital staff, patients’ relatives, and accompanying caregivers. This convenience sampling of controls may have introduced selection bias, including controls without lipedema volunteer bias. Furthermore, the high educational level of the control group limits the sample’s representativeness of the general female population and the generalizability of the findings. Third, most participants with lipedema had not previously received treatment, and the sample included no participants with lipolymphedema, restricting the generalizability of the results to women who have received treatment or developed lipolymphedema. Internal consistency was not assessed in this sample; therefore, reliability estimates from previous validation studies cannot confirm the reliability of the questionnaires in the present sample. Finally, although excluding participants with a previously diagnosed eating disorder reduced the potential influence of known eating disorders on the findings, this criterion may also have led to underestimation of the true burden of elevated orthorexic eating tendencies in an unselected lipedema population. Although the primary objective was to compare DOS scores between women with lipedema and controls, the original a priori sample-size calculation was based on the anticipated DOS–BSQ correlation rather than the primary between-group comparison. Therefore, the subsequent sensitivity analysis should be interpreted as a characterization of the detectable effect size of the achieved sample and not as a substitute for an a priori power calculation.

Future longitudinal studies should examine the temporal relationship between body shape concern and orthorexic eating, as well as changes in these tendencies following diagnosis or treatment. Larger, multicenter samples may allow subgroup analyses by lipedema type, morphological stage, disease duration, and pain intensity. Combining dimensional scales with structured clinical interviews will help determine the clinical significance of orthorexic eating tendencies.

## 5. Conclusions

Women with clinically diagnosed lipedema exhibited greater orthorexic tendencies, body shape concern, and psychological symptom levels than controls. When the variables examined were considered together, body shape concern was the only variable independently associated with DOS scores. The group difference in DOS scores is largely explained by the higher BSQ scores accompanying lipedema; this should not be interpreted as indicating that lipedema is unrelated to orthorexic tendencies. These findings provide a rationale for research assessing eating behavior alongside body image in lipedema; however, they do not demonstrate mediation, causality, or intervention efficacy.

## Figures and Tables

**Table 1:** Demographic and clinical characteristics of the study participants.

Variable	Controls (*n* = 44)	Lipedema (*n* = 44)	*p* Value
Age (years)	32.09 ± 8.62	35.41 ± 9.27	0.081 ^1^
BMI (kg/m^2^)	24.87 ± 4.32	25.71 ± 5.10	0.628 ^1^
Marital status			0.013 ^2^
Single/Divorced	20 (45.5%)	9 (20.5%)	
Married	24 (54.5%)	35 (79.5%)	
Education level			0.007 ^2^
Primary/High school	2 (4.5%)	11 (25.0%)	
University/Postgraduate	42 (95.5%)	33 (75.0%)	
Occupation			0.261 ^2^
Unemployed/Retired	16 (36.4%)	10 (22.7%)	
Office worker	16 (36.4%)	23 (52.3%)	
Manual worker	12 (27.3%)	11 (25.0%)	
Smoking			0.269 ^2^
Never/Former	38 (86.4%)	34 (77.3%)	
Current smoker	6 (13.6%)	10 (22.7%)	
Alcohol consumption			0.375 ^2^
None	26 (59.1%)	30 (68.2%)	
Social/Current	18 (40.9%)	14 (31.8%)	
Comorbidity			0.259 ^2^
None	37 (84.1%)	33 (75.0%)	
≥1 comorbidity	7 (15.9%)	11 (25.0%)	
Regular medication use			0.560 ^2^
No	38 (86.4%)	36 (81.8%)	
Yes	6 (13.6%)	8 (18.2%)	
Previous psychiatric diagnosis			0.725 ^2^
No	39 (88.6%)	40 (90.9%)	
Yes	5 (11.4%)	4 (9.1%)	

^1^ Mann–Whitney U test. ^2^ Chi-square test (or Fisher’s exact test where appropriate). Values are presented as mean ± standard deviation or *n* (%).

**Table 2:** Clinical characteristics of patients with lipedema (*n* = 44).

Variable	Value
Pain intensity (VAS)	5.57 ± 2.24
Disease duration	
Newly diagnosed	7 (15.9%)
<1 year	8 (18.2%)
1–3 years	19 (43.2%)
3–5 years	8 (18.2%)
>5 years	2 (4.5%)
Family history of lipedema	
Yes	22 (50.0%)
No	10 (22.7%)
Unknown	12 (27.3%)
Lipedema type	
Type I	8 (18.2%)
Type II	12 (27.3%)
Type III	24 (54.5%)
Lipedema stage	
Stage 1	14 (31.8%)
Stage 2	24 (54.5%)
Stage 3	6 (13.6%)
Previous treatment for lipedema	
No	39 (88.6%)
Yes	5 (11.4%)

Values are presented as mean ± SD or *n* (%). Family history was self-reported and categorized as yes, no, or unknown.

**Table 3:** Comparison of orthorexic tendency, body image dissatisfaction, anxiety, and depression between controls without lipedema and patients with lipedema.

Variable	Controls (*n* = 44)	Lipedema (*n* = 44)	Mean Difference (95% CI)	Cohen’s *d* (95% CI)	*p* Value
DOS	20.48 ± 5.05	25.07 ± 6.55	4.59 (2.11 to 7.07)	0.79 (0.35 to 1.22)	<0.001
BSQ	81.20 ± 32.00	111.89 ± 33.26	30.68 (16.85 to 44.51)	0.94 (0.50 to 1.38)	<0.001
HADS-A	6.66 ± 3.71	9.34 ± 3.48	2.68 (1.16 to 4.21)	0.75 (0.31 to 1.18)	0.001
HADS-D	4.39 ± 2.64	7.50 ± 3.65	3.11 (1.76 to 4.46)	0.98 (0.53 to 1.42)	<0.001

Values are presented as mean ± standard deviation (SD). Between-group comparisons were performed using the independent samples *t*-test. Mean differences are presented with 95% confidence intervals (CIs). Effect sizes are presented as Cohen’s *d* with 95% CI. DOS, Düsseldorf Orthorexia Scale; BSQ, Body Shape Questionnaire; HADS-A, Hospital Anxiety and Depression Scale–Anxiety; HADS-D, Hospital Anxiety and Depression Scale–Depression.

**Table 4:** Correlations between orthorexic tendency (DOS) and demographic, anthropometric, and psychological variables in controls without lipedema and patients with lipedema.

Variable	Controls Without Lipedema (r)	*p* Value	Lipedema (r)	*p* Value
Age	0.215	0.161	0.156	0.311
BMI	0.178	0.248	0.152	0.325
BSQ	**0.339**	**0.024**	**0.382**	**0.011**
HADS-A	0.118	0.445	0.081	0.603
HADS-D	0.064	0.678	0.155	0.314

Pearson correlation coefficients (r) are presented. DOS, Düsseldorf Orthorexia Scale; BMI, body mass index; BSQ, Body Shape Questionnaire; HADS-A, Hospital Anxiety and Depression Scale–Anxiety; HADS-D, Hospital Anxiety and Depression Scale–Depression. Statistically significant correlations are shown in **bold**.

**Table 5:** Multiple linear regression analysis of factors associated with Düsseldorf Orthorexia Scale scores.

Variable	B	SE	Standardized β	95% CI for B	*p* Value
Age, years	0.110	0.070	0.159	−0.029 to 0.248	0.120
BMI, kg/m^2^	0.028	0.135	0.021	−0.242 to 0.297	0.838
BSQ score	0.067	0.021	0.384	0.025 to 0.108	0.002
HADS-A score	−0.161	0.218	−0.099	−0.595 to 0.273	0.461
HADS-D score	0.229	0.224	0.129	−0.217 to 0.674	0.310
Lipedema group	1.875	1.406	0.151	−0.923 to 4.672	0.186

Model statistics: R^2^ = 0.287; adjusted R^2^ = 0.234; F(6,81) = 5.435; *p* < 0.001. B, unstandardized regression coefficient; SE, standard error; β, standardized regression coefficient; BMI, body mass index; BSQ, Body Shape Questionnaire; HADS-A, Hospital Anxiety and Depression Scale–Anxiety; HADS-D, Hospital Anxiety and Depression Scale–Depression.

## Data Availability

The datasets generated and analyzed during this study are available from the corresponding author upon reasonable request.

## Abbreviations

BMI: Body mass index; BSQ: Body Shape Questionnaire; DOS: Düsseldorf Orthorexia Scale; CI: Confidence interval; HADS: Hospital Anxiety and Depression Scale; HADS-A: HADS anxiety subscale; HADS-D: HADS depression subscale; SD: Standard deviation; STROBE: Strengthening the Reporting of Observational Studies in Epidemiology.
